# Posterior sagittal anorectoplasty in anorectal anomalies: clinical, manometric and profilometric evaluation

**DOI:** 10.1590/S1516-31802007000300007

**Published:** 2007-05-03

**Authors:** Pedro Félix Vital, José Luiz Martins, Fábio Luís Peterlini

**Keywords:** Child, Anal canal, Imperforate anus, Manometry, Fecal incontinence, Criança, Canal anal, Anus imperfurado, Manometria, Incontinência fecal

## Abstract

**CONTEXT AND OBJECTIVE:**

Anorectal malformations comprise a spectrum of anomalies that continue to be difficult to treat, even today. The aim was to evaluate the fecal continence of children who underwent posterior sagittal anorectoplasty due to anorectal malformations, via computerized anorectal manometry and profilometry.

**DESIGN AND SETTING:**

Prospective study at Universidade Federal de São Paulo.

**METHOD:**

82 patients (56.1% boys; 43.9% girls) of mean age 85.5 months were evaluated. They were divided into continent, partially continent and incontinent groups. Age, sex, manometric variables and profilometric parameters were studied. The results were statistically analyzed.

**RESULTS:**

Among the 82 patients, 37.8% were continent, 25.6% were partially continent and 36.6% were incontinent. The overall mean resting pressure was 22 mmHg, and the means for the continent, partially continent and incontinent groups were, respectively, 30.7 mmHg, 23 mmHg and 14.7 mmHg. The overall mean pressure response to voluntary contraction was 56 mmHg, and the means for the groups were 65.4 mmHg, 55.8 mmHg and 46.6 mmHg, respectively. The rectosphincteric reflex was absent in 82.9% of the cases. In the profilometry analysis for all patients together, blue (20 to 50 mmHg) and yellow (50 to 80 mmHg) were predominant, and there was a similar distribution for the continent and partially continent patients. However, among the incontinent patients, green (< 20 mmHg) and blue prevailed.

**CONCLUSIONS:**

Manometric and computerized profilometric analyses were an excellent method for postoperative evaluations on patients with intermediate and high anorectal anomalies, and for therapeutic planning.

## RESUMO

**CONTEXTO E OBJETIVO:**

As anomalias anorretais correspondem a um espectro de malformações de tratamento difícil mesmo nos dias de hoje. O objetivo foi avaliar crianças portadoras de anomalias anorretais altas e intermediárias, operadas pela anorretoplastia sagital posterior quanto à continência fecal através da manometria anorretal e profilometria computadorizadas.

**TIPO DE ESTUDO E LOCAL:**

Trabalho prospectivo, na Universidade Federal de São Paulo.

**MÉTODO:**

Avaliamos 82 pacientes agrupados em continentes, parcialmente continentes e incontinentes quanto a idade, sexo e variáveis padronizadas na manometria anorretal e profilometria. Os resultados foram analisados estatisticamente.

**RESULTADOS:**

Dos 82 pacientes 37,8% eram continentes, 25,6% parcialmente continentes e 36,6% incontinentes. A média da pressão de repouso à manometria anorretal foi de 22 mmHg, sendo entre os continentes, parcialmente continentes e incontinentes, respectivamente de 30,7 mmHg, 23 mmHg e 14,7 mmHg. A média da resposta pressórica à contração voluntária foi de 56 mmHg, sendo entre os continentes 65,4 mmHg, parcialmente continentes 55,8 mmHg e incontinentes 46,6 mmHg. O reflexo reto-esfincteriano encontrava-se ausente em 82,9% dos casos. Predominaram na profilometria as cores azul (20 a 50 mmHg) e amarela (50 a 80 mmHg), quando todo o grupo foi analisado conjuntamente, com padrão semelhante entre os continentes e parcialmente continentes; nos incontinentes, destacaram-se as cores verde (< 20 mmHg) e azul.

**CONCLUSÕES:**

A manometria anorretal computadorizada e a profilometria mostraram-se úteis na avaliação do comportamento pressórico esfincteriano, assim como no acompanhamento pós-operatório e planejamento terapêutico dos pacientes.

## INTRODUCTION

Posterior sagittal anorectoplasty, which was proposed by Alberto Peña and Pieter de Vries in 1982,^1^ revolutionized the surgical treatment of anorectal anomalies. This technique provided better understanding of the anatomical relationships between the structures involved in such malformations. Nonetheless, anorectal anomalies continue to be difficult to treat, even today.

## OBJECTIVE

The aim of this study was to evaluate patients with anorectal anomalies who underwent surgery using the technique of posterior sagittal anorectoplasty, from the point of view of fecal continence and pressure profile, using computerized anorectal manometry with continuous perfusion and profilometry.

## METHOD

Eighty-two children with anorectal anomalies were evaluated within the Pediatric Surgery Division, Department of Surgery, Universidade Federal de São Paulo — Escola Paulista de Medicina (Unifesp-EPM), between 2001 and 2004. Among these, there were 45 cases of high anorectal anomaly and 37 of intermediate anomaly. The children's ages were between 12 and 204 months, and there were 46 males and 36 females. All of them were at a late postoperative stage, i.e. more than nine months after the surgical procedure to correct anorectal malformation.

These patients were studied prospectively from the clinical point of view, and by means of computerized anorectal manometry using the perfusion method and profilometry, with regard to their capacity for fecal continence.

### Surgical procedures

All the patients had initially undergone temporary colostomy in the descending colon with two openings (proximal and distal), 24 hours after their birth. Posterior sagittal anorectoplasty was performed subsequently, at a mean of four months of life. This procedure comprised a sagittal incision between the coccyx and perineum that extended to the parasagittal fibers of the external sphincter muscle of the anus and the sphincter muscle complex. Then full dissection was done using a electrical muscle stimulator to identify the different parts of the muscular sphincter complex.

The rectal pouch was identified and mobilized. It was then dissected, opened and enveloped within muscle tissue of the sphincter muscle complex. Following this, the anorectoplasty was completed.

Anal dilatation procedures were begun two weeks after the anorectoplasty, and lasted for up to eight weeks. They were continued until adequate canal caliber was achieved (14 mm), when the colostomy could be closed and the intestinal lumen reconstructed.

### Postoperative evaluation

Six months after the reconstruction of the intestinal transit, the patients were evaluated clinically and by means of anorectal manometry and profilometry.

### A. Evaluation of fecal continence

The patients were divided into three groups:

- Continent: patients who evacuated once or twice a day, with feces of normal appearance and consistency, without episodes of escape and without staining their underwear. Rectal examination showed good contractility of the sphincter muscle complex;
- Partially continent: individuals who evacuated three to five time a day, with feces of pasty consistency and episodes of escapes, and frequent soiling of underwear. The anus had a regular appearance upon inspection, often with small areas of prolapsed mucosa of dirty appearance. Rectal examination showed regular contractions only in the upper and lower portions of the sphincter muscle complex;
- Fecally incontinent: patients who evacuated more than five times a day, with feces of liquid consistency and constant total fecal loss. The anus had an abnormal appearance, either with a pronounced opening or with significant prolapsed mucosa, with feces visibly leaking out. Rectal examination showed weak contractions, or their absence, in all segments of the sphincter muscle complex.

### B. Evaluation by means of computerized anorectal manometry using the perfusion technique

A flexible catheter of four millimeters (mm) in diameter was utilized, with four radially-arranged orifices and a lumen to enable inflation of a latex balloon with a maximum capacity of 200 milliliters (ml), at its tip. The following variables were evaluated during the examination: resting pressure (RP), length of the anal canal, sphincter pressure response to coughing (CP), pressure response to voluntary contraction (VCP), maximum pressure obtained on the pressure curve (MP), pressure response during perianal stimulation (PAS), pressure curve analysis and rectosphincteric reflex (RSR). Three-dimensional graphics of the anorectal canal were produced from the tracings relating to the pressure curve (profilometry), to establish indices for total asymmetry, anorectal canal segment asymmetry and rectal volume (cm x cmHg^2^).

The catheter was connected to a four-channel pressure receptor, with a pneumatic CO^2^ system that kept the infusion pressure at one atmosphere (atm), with continuous flow of distilled water at the rate of 0.56 milliliters/minute (ml/min).^2,3^

The pressure data obtained were amplified, and the results were recorded and analyzed with the aid of the Proctomaster software (Dynamed; Dynapack MPX 850), with pressures presented in millimeters of mercury (mmHg).

After identifying the region of greatest pressure, the catheter was reintroduced in such a way as to position the perfusion orifices within this segment of greatest pressure, to make recordings, and was then fixed there.

**Figure 1:**
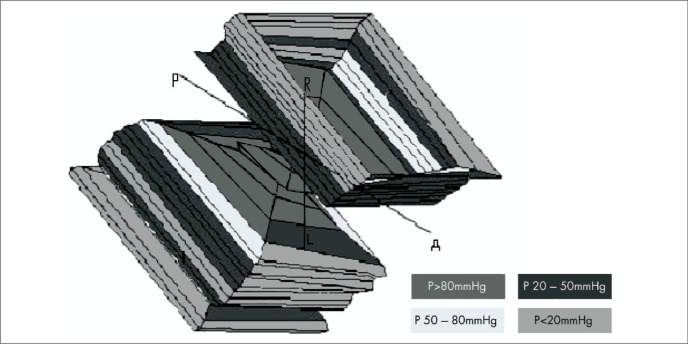
Profilometry: computer graphic of the pressure curves in the anal canal, obtained using continuous flow computerized anorectal manometry in children who underwent posterior sagittal anorectoplasty. Example of sphincter muscle complex with adequate pressure profile in anorectal canal. **A** = anterior; **P** = posterior; **R** = right; **L** = left.

From the tracings acquired in building up pressure curves from the four perfusion channels, obtaining pressure readings from the four quadrants, the computer program generated three-dimensional tracings of the anorectal canal. This made it possible to study the total and segmental asymmetry indices for this canal, the rectal volume (cm x cmHg^2^), and also the pressure distribution on the anorectal wall. The latter was translated via the program into a sequence of colors corresponding to pressures, on a previously established scale (green: < 20 mmHg; blue: 20-50 mmHg; yellow: 50-80 mmHg; red: > 80 mmHg) (Figure 1).

For the quantitative variables, means and medians were utilized to summarize the information, and standard deviations, minimums and maximums to indicate the variability of the data. Absolute (n) and relative frequencies (%) were presented for the non-parametric data, and also the following statistical tests: chi-squared, analysis of variance (ANOVA), Tukey, non-parametric Kruskal-Wallis, non-parametric Mann-Whitney and Student's t.

For all of these, the significance level adopted was 5%. Thus, associations or statistical differences between groups were considered valid when the results from the tests presented values less than 0.05 (p-value less than or equal to 0.05).

## RESULTS

The numbers of patients in the three categories of fecal continence (continent, partially continent and incontinent) were similar, as shown in Table 1. There was predominance of high anorectal anomalies among the incontinent patients, and this is shown in Table 2. The means, medians and standard deviations of quantitative variables for all 82 patients are presented in Table 3.

**Table 1.** Distribution profile of fecal continence in sample of children who underwent posterior sagittal anorectoplasty

	n	%
Continent individuals	31	37.8
Partially continent individuals	21	25.6
Incontinent individuals	30	36.6
**Total**	**82**	**100**

**Table 2.** Distribution of high and intermediate anorectal anomalies in the continent, partially continent and incontinent groups of children who underwent posterior sagittal anorectoplasty

Type of anorectal anomaly	Continent		Partially continent		Incontinent	
	n	%	n	%	n	%
High	12	38.7	11	52.4	22	73.3
Intermediate	19	61.3	10	47.6	8	26

**Table 3.** Presentation of the means, medians and standard deviations for the variables among children who underwent posterior sagittal anorectoplasty: age, resting pressure (RP), voluntary contraction pressure (VCP), anal canal length, total asymmetry index (TAI), segmental asymmetry index (SAI) and rectal volume (RV), from anorectal manometry (n = 82)

	Mean	Median	Standard deviation	Minimum	Maximum
Age (months)	85.5	72.0	50.6	12.0	204.0
RP (mmHg)	22.9	20.0	10.3	7.0	47.0
VCP (mmHg)	56.0	53.0	18.3	16.0	106.0
Anal canal (cm)	2.5	2.0	0.9	1.0	5.0
TAI (%)	29.8	28.5	10.3	11.1	60.0
SAI (%)	25.2	24.0	12.1	2.3	54.7
RV (cm x cmHg^2^)	280.3	151.3	382.9	9.2	1349.0

The patient distribution was analyzed in relation to sex, presence or absence of rectosphincteric reflex, the behavior of the pressure curves with regard to shapes and pressure levels, and profilometry mapping, for the continent, partially continent and incontinent groups of patients. The results from this are presented in Table 4.

**Table 4.** Comparisons between the continent, partially continent and incontinent groups of children who underwent posterior sagittal anorectoplasty, in relation to the qualitative variables of sex, rectosphincteric reflex (RSR), pressure curve shape, pressure curve level and profilometry

		Group						Descriptive level (p-value)
		Continent		Partially continent		Incontinent		
		n	%	n	%	n	%	
Sex	male	12	38.7%	11	52.4%	23	76.7%	**0.011**
	female	19	61.3%	10	47.6%	7	23.3%	
RSR	absent	20	64.5%	20	95.2%	28	93.3%	**0.003**
	present	11	35.5%	1	4.8%	2	6.7%	
Pressure curve shape	abnormal	12	38.7%	10	47.6%	23	76.7%	**0.009**
	normal	19	61.3%	11	52.4%	7	23.3%	
Pressure curve level	low	3	9.7%	7	33.3%	17	56.7%	**0.003**
	normal	16	51.6%	8	38.1%	9	30.0%	
	High	12	38.7%	6	28.6%	4	13.3%	
Profilometry	Green	3	9.7%	2	9.5%	13	43.3%	**0.003**
	Blue	15	48.4%	12	57.1%	15	50.0%	
	Yellow	12	38.7%	5	23.8%	2	6.7%	
	Red	1	3.2%	2	9.5%			

The distribution by age groups, mean resting pressure, voluntary contraction pressure, anal canal length, total and segmental anorectal canal asymmetry indices and rectal volume was evaluated separately for the continent, partially continent and incontinent groups of patients. These results are presented in Table 5.

**Table 5.** Comparison between the continent, partially continent and incontinent groups of children who underwent posterior sagittal anorectoplasty and the means, medians and standard deviations for the quantitative variables of age, resting pressure, voluntary contractions, anal canal length, total and segmental asymmetry indices and rectal volume

		Group			Descriptive level (p-value)
		Continent	Partially continent	Incontinent	
Age (months)	Mean	67.3	80.1	108.0	
	Median	48.0	75.0	102.0	
	Standard deviation	50.5	41.7	49.2	**0.005**
	Minimum	12.0	24.0	23.0	
	Maximum	204.0	168.0	192.0	
Resting pressure (mmHg)	Mean	30.7	23.0	14.7	
	Median	30.0	22.0	12.5	
	Standard deviation	9.9	6.8	5.2	**0.000**
	Minimum	16.0	15.0	7.0	
	Maximum	47.0	44.0	26.0	
Voluntary contraction pressure (mmHg)	Mean	65.4	55.8	46.6	
	Median	64.0	52.0	45.5	
	Standard deviation	18.7	16.0	14.3	**0.000**
	Minimum	31.0	22.0	16.0	
	Maximum	106.0	90.0	78.0	
Anal canal (cm)	Mean	2.5	2.4	2.5	
	Median	2.0	2.0	2.0	
	Standard deviation	0.8	0.9	1.1	0.916
	Minimum	1.0	1.0	1.0	
	Maximum	4.0	4.0	5.0	
Total asymmetry index (%)	Mean	30.0	28.3	30.7	
	Median	28.0	25.5	30.5	
	Standard deviation	9.3	11.6	10.6	0.724
	Minimum	11.1	11.6	12.6	
	Maximum	51.0	53.4	60.0	
Segmental asymmetry index (%)	Mean	23.4	23.6	28.1	
	Median	22.0	22.7	28.1	
	Standard deviation	11.0	11.6	13.2	0.257
	Minimum	7.3	2.3	5.0	
	Maximum	53.0	48.4	54.7	
Rectal volume (cm x cmHg^2^)	Mean	297.4	349.2	167.1	
	Median	205.7	143.8	93.0	
	Standard deviation	278.5	410.5	254.9	**0.023**
	Minimum	14.2	11.7	9.2	
	Maximum	1339.0	1349.0	1349.0	

It was observed that there were differences between the groups with regard to the variables of age, resting pressure and voluntary contraction pressure. To identify which groups presented differences between each other, multiple comparisons were performed, as shown in Table 6.

**Table 6.** Multiple comparisons to identify statistical significance between pairs, in relation to age (months), resting pressure (mmHg) and voluntary contraction pressure (mmHg) among children who underwent posterior sagittal anorectoplasty

Groups compared	Age p-value	Resting pressure p-value	Voluntary contraction pressure p-value
Continent and partially continent	0.613	**0.006**	0.106
Continent and incontinent	0.004	**0.000**	**0.000**
Partially continent and incontinent	0.108	**0.000**	0.130

It was observed from the results above that there were differences in age between the continent and incontinent patients, such that the incontinent patients on average presented greater ages. It was also seen that there were differences in resting pressure between all the groups, such that the incontinent patients presented the lowest pressure values, followed by the partially continent patients and then the continent patients, with the greatest mean values. Furthermore, there were differences in voluntary contraction pressure between the continent and incontinent patients, such that the incontinent patients on average presented lower values.

Table 7 presents the linear correlations between the variables of age, resting pressure, voluntary contraction pressure and anal canal length. The index utilized was Pearson's correlation coefficient, varying from - 1 to + 1, which indicates stronger correlations as the values come closer to the extremities. The greatest value found for Pearson's correlation coefficient was 0.60, thereby showing that there was a tendency towards a positive linear association between the resting pressure and the voluntary contraction pressure, i.e. the greater the value of the resting pressure was, the greater the voluntary contraction pressure. The correlations between the other variables were low in magnitude. There was no great correlation between age and any other variable.

**Table 7.** Linear correlations between the quantitative variables of age, resting pressure (RP), voluntary contraction pressure (VCP), anal canal length and rectal volume among children who underwent posterior sagittal anorectoplasty

	Age (months)	RP	VCP	Anal canal
Age (months)	1			
RP (mmHg)	-0.28	1		
VCP (mmHg)	-0.21	**0.60**	1	
Anal canal (cm)	-0.13	0.14	0.21	1
RV (cm x cmHg^2^)	-0.17	0.19	0.26	0.08

## DISCUSSION

Despite advances in surgical techniques, the preservation of the mechanisms essential for fecal continence and voluntary control over evacuations is often deficient following surgical treatment of intermediate and high anorectal anomalies.^4–6^

Various researchers^7–14^ have done electrophysiological anorectal evaluations by means of anorectal manometry. This has specific applications in cases of intestinal constipation; fecal incontinence; post-traumatic, postoperative and infectious sequelae of the anorectal region; and, especially, in postoperative evaluation of anorectal malformations.

We had overall predominance of male individuals (56.1%) among our patients. However, when they were divided into the three specific groups of continent, partially continent and incontinent patients, the majority of the continent individuals were female (61.3%; p = 0.011).

The median age allowed us to make a reliable assessment of the degree of continence, in relation to when continence is physiologically acquired. In general, this is at preschool age, even though some authors^15,16^ have believed otherwise and have published studies on anorectal anomalies using anorectal manometry among premature and low birth weight children, while obviously making allowances for their physiological characteristics and the absence of systemic manifestations that would interfere in the results.

We found continence in 37.8% of the cases in our sample, partial continence in 25.6% and incontinence in 36.6%. We have attributed an important role to the resting pressure, in the process of acquiring continence, although we are aware that, especially in cases of high anorectal anomalies, this variable is related to the presence of a striated muscle complex, particularly the external anal sphincter muscle.^17–19^

Muscle activity was studied in our sample by means of the behavior of the pressure curves, which were found to present normal shapes and pressure intensity levels in a little under half of the cases: 45.1% and 40.2%, respectively. When these results were compared with the capacity for continence among the 82 children evaluated, we observed that the sum of the percentages of continent and partially continent patients (63.4%) practically coincided with the sum of the percentages of curves with normal and high pressure intensity levels (67%). This association could also be seen between the incontinent individuals (36.6%) and the percentage of curves with a pressure intensity level of low power (32.95%).

These graphical representations made it possible to identify the patients with amputated curves, thus revealing a compromised external anal sphincter muscle. Likewise, large amplitude curves represented postoperative scar stenosis, and tracings with rectified ascending curves denoted irregularities of the puborectal bundle of the levator ani muscle, among others.

Curves of low-power pressure intensity were related to patients with low capacity for sphincteric contractions, and these were thus children with low potential for continence.

Low-amplitude curves with low pressure intensity levels led us to anorectal anomalies of greater therapeutic complexity, with deficient or even non-existent striated sphincteric musculature, or to a reconstruction with inadequate localization of the neo-anus during the posterior sagittal anorectoplasty. This pressure curve pattern was found in 9.7% of the continent patients, 33.3% of the partially continent and 56.7% of the incontinent patients.

There were curves of high pressure intensity level in 13.3% of the patients in the incontinent group. In fact, there were patients who frequently soiled their underwear with feces. They presented absence of effective evacuations that was probably due to postoperative scar stenosis, with or without associated inefficiency in anal dilatation sections. We found high-amplitude curves with high pressure levels for these children, and large rectal volumes. These children could be considered to have pseudo-incontinence, which would probably present therapeutic possibilities.

The mean resting pressure observed was 22.9 mmHg and the mean voluntary contraction pressure was 56 mmHg. These values, despite being within the normal limits in relation to those presented in manometric studies, were always close to the lower limit. The mean for the voluntary contraction pressure was practically twice the resting pressure, which is generally expected when continence capacity is investigated.

Measurement of the pressure in the anal canal has been a useful and commonly used way of assessing sphincter function and providing valuable information.^20–24^ Our analysis of the mean resting pressure showed that the greatest pressure levels were among the continent patients (30.7 mmHg), in relation to the partially continent (23 mmHg) and incontinent patients (14.7 mmHg), and these pressure differences were very representative from a statistical point of view (p = 0.001).

In a general manner, these mean resting pressures were low in the three groups evaluated, and similar values have been found by other authors.^25^ It is our understanding that the threshold levels for resting pressure among patients with high and intermediate anorectal anomalies are a reflection of the abnormalities in their anatomical development. Likewise, lesions of the neuromuscular structures essential for fecal continence that occur during surgical correction may interfere in the short and long-term prognosis.

However, we found that threshold pressure values could provide states of full continence, partial continence or incontinence, without interference from other elements in this process, for example fecal consistency.

There was a tendency towards linear growth when we correlated resting pressures and voluntary contraction pressures, which makes us believe even more in the active participation of the external anal sphincter in maintaining the resting pressure among patients treated for high and intermediate anorectal anomalies.

In studying the behavior of the pressure response to voluntary contraction, which is generated primarily by the external sphincter muscles of the anus and the levator ani muscle, with major participation by its puborectal sling,^26^ we found higher values among the continent patients (65.4 mmHg) than among the partially continent (55.8 mmHg) and incontinent patients (46.6 mmHg), with p < 0.0001.

This statistical significance between the continent and incontinent groups of patients was large. The partially continent individuals presented mean voluntary contraction pressures that were very close to those of the continent patients, which makes us optimistic about their prognoses, with the application of appropriate exercises to improve their fecal continence.

Reflex relaxation of the internal sphincter muscle of the anus induced by rectal distension is associated with suppression of the electrical oscillations in this sphincter.^27–29^ Rectosphincteric reflex was found in 17.1% of the 82 patients examined. When the patient groups were analyzed separately, we observed that rectosphincteric reflex was detected in 35.5% of the continent children, 4.8% of the partially continent and 6.7% of the incontinent children, i.e. with very distinct distribution between these groups.

The presence of rectosphincteric reflex was attributed to the fact that we had patients with anorectal anomalies of intermediate type in our sample. The internal anal sphincter may be present in such patients. The presence of rectosphincteric reflex in patients with high anorectal anomalies may be a reactional response by the external sphincter muscle to favor the passage of feces, most probably in a conscious manner. In some other cases, it may be an artifact of the examination. Thus, it remains unclear whether or not rectosphincteric reflex is the best indicator for anorectal function following surgery to correct anorectal anomalies.^30^

The lengths of the anal canal in the continent, partially continent and incontinent groups of patients presented a small range of means, from 2.4 to 2.5 centimeters. According to the results presented, the participation of the length of the functional anal canal in the capacity for fecal continence among patients with high and intermediate anorectal anomalies following posterior sagittal anorectoplasty did not show statistical significance between the groups evaluated. Some researchers have attributed to the length of the functional anal canal an active participation in maintaining continence, either separately or in association with other anatomical elements.^31,32^

We believe that computerized profilometry is one of the major innovatory contributions towards patient care following posterior sagittal anorectoplasty. It is capable of providing reliable information regarding the three-dimensional topography of the anorectal canal, and also regarding the distribution of the pressures involved in the process of acquiring anorectal fecal continence, thus greatly assisting in the clinical conduct administered to such patients.^33–36^

In evaluating the 82 patients in the sample presented, we found predominance of the colors blue and yellow (74.4%), which represents a predominance of 20-50 mmHg and 50-80 mmHg. These mathematical values represented pressures of between 20 and 80 mmHg that were observed at the anorectal walls. The highest pressure levels were correlated with the anal canal. This percentage was greater than the sum of the percentages of continent and partially continent patients (63.4%). In other words, the graphical presentations showed better results than did the clinical behavior, which demonstrates potential for improving the state of continence, if appropriate training for this purpose is begun. Another element to be highlighted is the possibility for change in this color pattern, especially among the partially continent patients, who might start to present the pattern for continent patients after undergoing training through an appropriate exercise program.

The means for total and segmental anorectal asymmetry were similar between the three groups of patients (continent, partially continent and incontinent). These values were close to those considered normal in the literature. These data show us that the surgery was well executed from a technical point of view, although it is evident that, in addition to well-performed surgery, other factors are also involved in the state of continence following posterior sagittal anorectoplasty, such as the anatomy of the malformation.

We found a mean rectal volume of 280 cm x cmHg^2^. For the continent, partially continent and incontinent groups of patients, the mean values found were 279.4, 349.2 and 167.1 cm x cmHg^2^, respectively. The differences were statistically significant (p = 0.023), with the biggest difference between the continent and incontinent patients.

In our sample, the partially continent patients presented greater rectal volumes than did the other groups. This pattern portrays mixed behavior, with characteristics of the two end groups (continent and incontinent). On the other hand, patients with large rectal volumes, abnormal pressure curves of high intensity and large amplitudes, led us to a diagnosis of hypertonic sphincter, with fecal retention and fecal escapes at inappropriate moments, thus characterizing a condition of pseudo-incontinence.^37–40^

Posterior sagittal anorectoplasty has been shown to be an excellent technique for treating anorectal anomalies, and good results without greatly compromised neuromuscular structures have been achieved. Nonetheless, many patients may continue to present functional problems following surgery, reflected through varying degrees of fecal escape and constipation.^41–45^

## CONCLUSIONS

Computerized anorectal manometry and profilometry are very useful methods for physiologically evaluating the anorectal region of children who have undergone surgery to correct anorectal anomalies. They are also very useful for postoperative follow-up and therapeutic planning, with objective and efficacious criteria.
